# Thiophene Stability in Photodynamic Therapy: A Mathematical Model Approach

**DOI:** 10.3390/ijms25052528

**Published:** 2024-02-21

**Authors:** Jackson J. Alcázar

**Affiliations:** Centro de Química Médica, Facultad de Medicina Clínica Alemana, Universidad del Desarrollo, Santiago 7780272, Chile; jackson.alcazar@udd.cl

**Keywords:** safe PDT, efficient PDT, thiophene-containing photosensitizer, singlet oxygen, conceptual DFT

## Abstract

Thiophene-containing photosensitizers are gaining recognition for their role in photodynamic therapy (PDT). However, the inherent reactivity of the thiophene moiety toward singlet oxygen threatens the stability and efficiency of these photosensitizers. This study presents a novel mathematical model capable of predicting the reactivity of thiophene toward singlet oxygen in PDT, using Conceptual Density Functional Theory (CDFT) and genetic programming. The research combines advanced computational methods, including various DFT techniques and symbolic regression, and is validated with experimental data. The findings underscore the capacity of the model to classify photosensitizers based on their photodynamic efficiency and safety, particularly noting that photosensitizers with a constant rate 1000 times lower than that of unmodified thiophene retain their photodynamic performance without substantial singlet oxygen quenching. Additionally, the research offers insights into the impact of electronic effects on thiophene reactivity. Finally, this study significantly advances thiophene-based photosensitizer design, paving the way for therapeutic agents that achieve a desirable balance between efficiency and safety in PDT.

## 1. Introduction

Photodynamic therapy (PDT) is an emergent and actively researched therapeutic method [1,2,3]. Its sophisticated mechanism combines light, photosensitizers, and oxygen to produce cytotoxic reactive oxygen species (ROS) [4,5,6,7], such as singlet oxygen (^1^O_2_) [8]. The success of PDT hinges on the dual mechanisms of ROS generation—Type I and Type II processes. Type I involves electron transfer leading to radical species formation, while Type II predominantly generates singlet oxygen through energy transfer [4,5]. These processes are crucial for therapeutic efficacy, depending on both the abundant generation of these reactive species and the resilience of photosensitizers against ROS-induced oxidation [9,10,11,12]. Concurrently, the judicious selection of photosensitizers capable of self-degrading after treatment through oxidation by the produced ROS addresses crucial safety concerns. This self-degradation minimizes the risks of prolonged photosensitivity, underscoring the importance of meticulously adjusting the oxidative reactivity of photosensitizers. Such fine-tuning is essential for achieving an optimal equilibrium between the therapeutic effectiveness and safety of PDT, thereby enhancing its application as a minimally invasive and targeted cancer treatment strategy [13,14,15,16,17,18,19].

Shifting focus to the structural aspect, thiophene and its derivatives have emerged as strong candidates. They have gained significant attention as potential building blocks in the design of photosensitizers, primarily due to their electron-donating ability [20], their enhanced π-conjugation [21,22,23,24,25,26,27,28,29], and their unique photophysical and photochemical properties [30,31,32,33]. In the field of theranostic applications, these derivatives are particularly vital, encompassing the latest advancements in photosensitizers based on aggregation-induced emission luminogens (AIEgens) [14,34,35,36,37,38,39,40,41,42]. Photosensitizers containing thiophene are notable for their significant red-shifts in absorption and emission wavelengths [38,39,40,43], which is advantageous for deeper tissue penetration in therapeutic applications. Their enhanced intersystem crossing ability [44,45] ensures a higher yield of triplet-state photosensitizers, which is key for ROS generation [39,46]. Moreover, the incorporation of thiophene moieties in photosensitizers has enabled targeted action on specific cellular components, thereby enhancing the specificity of PDT [47,48].

In the context of the type II mechanism, an ideal photosensitizer would generate ^1^O_2_ while also being resistant to oxidative degradation. High stability in a photosensitizer can yield remarkable treatment efficiency, as it stays predominantly focused on its photodynamic role [49]. In opposition, the advantage of self-degradable photosensitizers lies in their post-treatment safety, as their oxidative breakdown facilitates removal from the body [13]. Furthermore, in scenarios where the photosensitizer accumulates significantly more in the tumor than in healthy tissue, photodegradation can aid in minimizing collateral damage during treatment [50,51]. Therefore, finding the right balance between stability and self-degradability is essential for optimizing both the efficiency and safety of the treatment. The inherent reactivity of thiophene toward singlet oxygen (^1^O_2_) can lead to the self-degradation of thiophene-based photosensitizers [52]. In this regard, a moderate reactivity toward ^1^O_2_ could be a critical attribute for achieving an optimal balance between safety and photodynamic efficiency.

Historically, research on thiophenes has been predominantly focused on petrochemical derivatization processes, particularly in the context of asphaltenes [53,54]. Their potential in the medical field, especially in PDT, has been largely overlooked [55,56,57]. This lack of recognition might account for the limited exploration of the reactivity of thiophene-containing compounds in the presence of singlet oxygen. To address this underexplored aspect, the application of Density Functional Theory (DFT) [58] within a theoretical framework emerges as a promising approach. This methodology could offer deeper insights into both the reactivity and practical applications of these compounds in PDT.

Building on this understanding, we aim to further advance the field by developing a mathematical model that forecasts the reactivity landscape of thiophene and its derivatives against ^1^O_2_. Using genetic programming [59,60], transition state theory [61,62], and the conceptual foundations of DFT [63,64,65,66,67,68], our approach seeks to provide a comprehensive framework for our model. This framework is meant to not only clarify the stability of thiophene-based photosensitizers but also to pave the way for their rational design, ensuring an optimal balance between efficiency and safety in PDT.

## 2. Results and Discussion

### 2.1. Mechanism and Theory Level

**Scheme 1:**
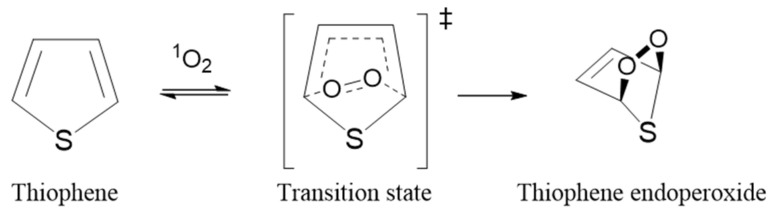
Illustration of the rate-determining step for singlet-oxygen oxidation of thiophene. The double dagger symbol (‡) denotes the transition state of reaction.

The oxidation reaction mechanism of thiophenes by singlet oxygen was theoretically investigated by Song et al. [69]. They reported the concerted [2 + 4] cycloaddition as the most favorable pathway, leading to the observed final product through the formation of an endoperoxide intermediate (see Scheme 1) [52,70]. The exploration of the reactivity of thiophenes in this study was structured based on this mechanism.

Table 1 reveals ωB97X-D3/def2-TZVP to be the level of theory that most accurately replicates the behavior of the eight relative rate constants as experimentally reported by van Tilborg [52]. This is further evidenced by the robust Pearson correlation coefficient squared (r^2^) of 98% associated with the ωB97X-D3/def2-TZVP level of theory, emphasizing its reliability and precision in modeling the reactivity changes in thiophenes. The functional ωB97X-D3 was selected for its established efficacy in predicting reaction barrier heights and thermodynamic properties [67].

**Figure 1:**
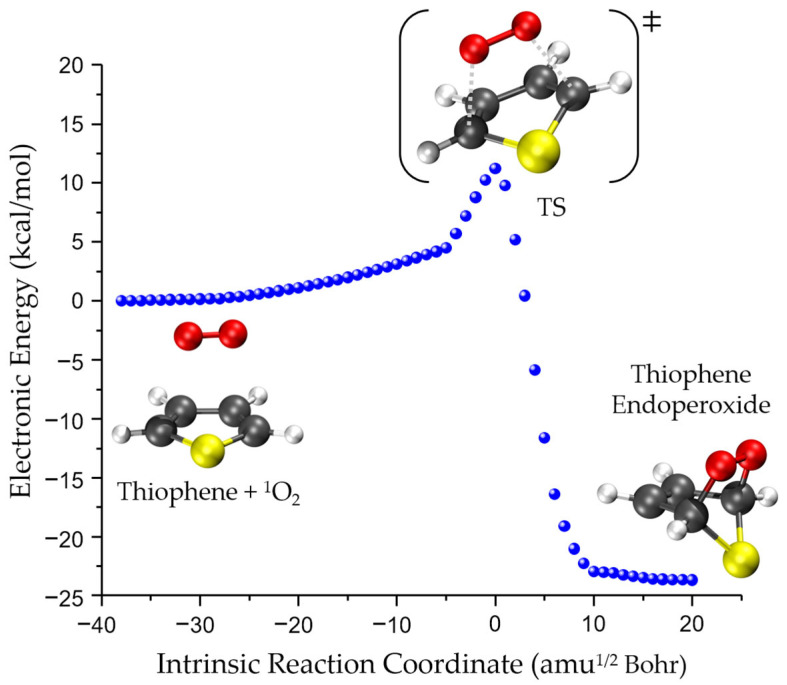
Intrinsic reaction coordinate (IRC) for the initial step of the thiophene oxidation reaction with singlet oxygen calculated using the ωB97X-D3/def2-TZVP level of theory. Depiction of thiophene and molecular oxygen in the Licorice model, represented with IUPAC color standards: sulfur (yellow), carbon (gray), and hydrogen (white) illustrate the structure of thiophene, while molecular oxygen is shown as connected red spheres.

Assessing the kinetics of these reactions in experimental settings presents significant limitations. In every instance, kinetic characterizations were systematically executed within the framework of transition state theory. The identification of each transition state was achieved by discerning the unique imaginary frequency, accompanied by a thorough Intrinsic Reaction Coordinate (IRC) analysis, as exemplified and illustrated in Figure 1.

### 2.2. The Role of Mulliken Electronegativity

**Scheme 2:**
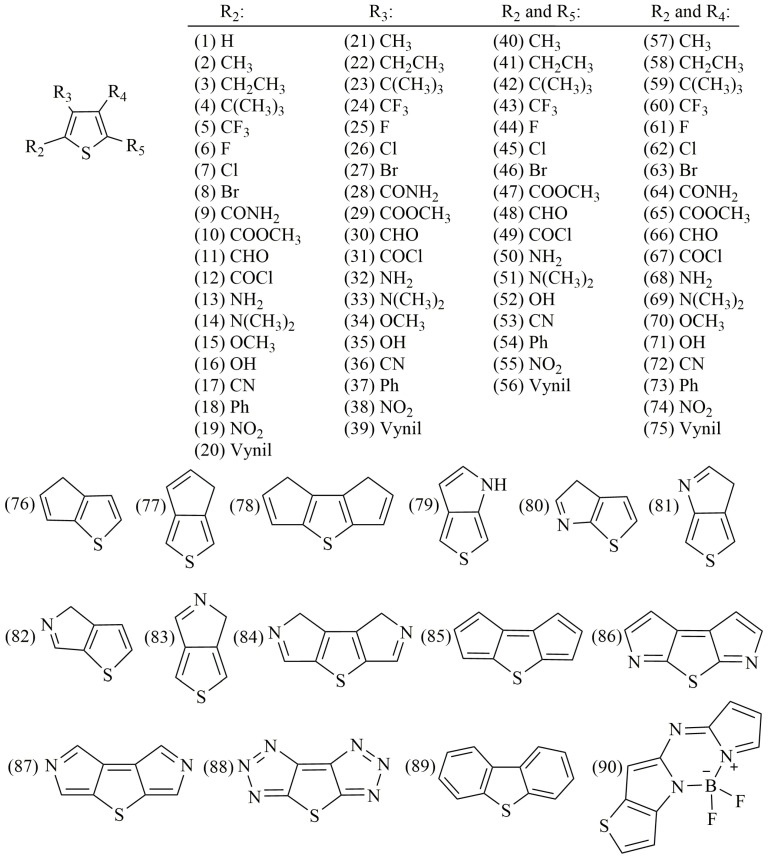
Structural modifications of thiophene designed to assess the influence of substituents and of the expansion of the thiophene scaffold on singlet oxygen oxidation kinetics.

To evaluate the influence of structural modifications on the reactivity of the thiophene scaffold in the presence of singlet oxygen, a comprehensive array of 90 derivatives was considered, encapsulating electronic effects such as inductive effect, resonance, and hyperconjugation, along with effects attributable to the structural ring expansion of the thiophene core. This approach enabled the analysis of thiophene reactivity in response to structural modifications. Derivatives are detailed in Scheme 2.

**Scheme 3:**
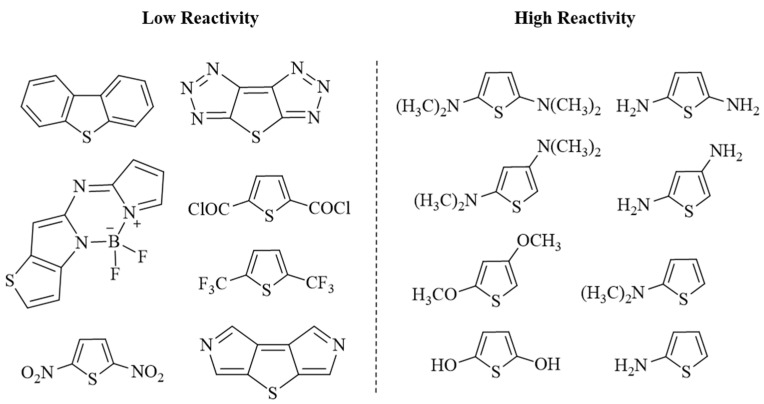
Least reactive and most reactive thiophene structures from the studied set.

The findings indicate that thiophene derivatives with substitutions at the alpha carbons to sulfur exhibit significant variations in rate constants compared to the unmodified thiophene. These variations in reactivity are markedly influenced by the nature of the substituents: electron-withdrawing groups are associated with lower reactivity, whereas electron-donating substituents lead to higher reactivity, as illustrated in Scheme 3.

To quantify the substituent effect, CDFT quantities were calculated, revealing that Mulliken electronegativity, χ_M_, correlates significantly with reactivity changes in the evaluated thiophenes.

**Figure 2:**
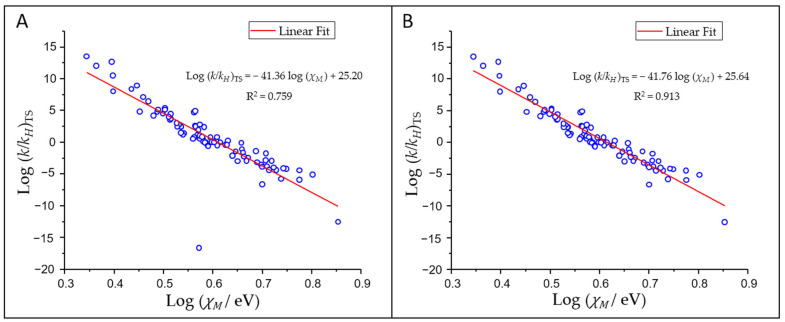
Plot of calculated logarithms of rate constant ratios (log (*k/k_H_*))_TS_ for singlet-oxygen oxidation of thiophenes versus logarithm of Mulliken electronegativity (log ($\chi_{M}$)). The log (*k/k_H_*) values are calculated in a vacuum using transition state theory and the ωB97X-D3/def2-TZVP level of theory. The log ($\chi_{M}$) values were obtained from conceptual DFT calculations carried out on the reactants. (**A**) Data for all 90 compounds analyzed. (**B**) Data after excluding compound **89**, highlighting the trend without the influence of the outlier.

As illustrated in Figure 2A, the logarithm of Mulliken Electronegativity (log (χ_M_)) exhibited a correlation coefficient (R^2^) of 0.759 with respect to the logarithms of rate constant ratios (log (*k*/*k*_H_)) computed for all 90 compounds. This correlation was notably enhanced (R^2^ = 0.913) upon the exclusion of the outlier: compound **89** (dibenzothiophene). The integrity of the calculations and the structural configuration for compound **89** were scrutinized without identifying any anomalies. Therefore, this deviation may arise from variations in π-conjugation configurations across the central ring, which arise when fused with six-membered rings as opposed to five-membered ones. Mulliken electronegativity cannot adequately describe the aforementioned structure, and the homogeneity of the data set is lost. To accurately include compound **89**, a more nuanced model must be considered.

Given that $\chi_{M}$ can demonstrate unusual reactivity patterns in thiophenes, it should be considered a rough indicator that highlights the effect of substituents on the overall reactivity trends in thiophenes. Higher electronegativity within thiophene-containing compounds correlates with decreased reactivity toward oxidation by singlet oxygen. These findings are in complete alignment with those reported by van Tilborg [71], who concluded that electron-withdrawing groups reduce thiophene reactivity in the presence of singlet oxygen. This underscores the quantitative significance of electron-withdrawing phenomena through Mulliken electronegativity, a concept that has been endorsed by Taft [71] and has recently been deemed essential in developing a predictive model for pK_a_ values [72].

In the context of PDT, we infer thiophene-containing photosensitizers with lower χ_M_ will exhibit greater resilience against oxidation by singlet oxygen. Conversely, thiophene-containing photosensitizers with higher $\chi_{M}$ could likely be categorized within the safer spectrum of PDT agents.

### 2.3. Reactivity Prediction Model

Determining the oxidation rate constants of thiophene-containing photosensitizers through the elucidation of transition states, and their corresponding thermodynamic properties, demands extensive computational resources that often exceed the realms of feasibility for many researchers.

Consequently, the application of transition state theory for predicting the reactivity of emergent photosensitizing agents may not be a feasible strategy for practitioners outside the specialized domain. An aspirational yet more accessible alternative would involve deducing the reactivity directly from the intrinsic molecular architecture of the photosensitizer, thereby avoiding the need for extensive analysis of the transition state and thermodynamic properties.

To address this challenge, we have integrated the principles of conceptual DFT with the robust methodology of genetic programming to facilitate symbolic regression analyses. This synergistic application has culminated in the derivation of a mathematical model capable of simulating the reactivity trends obtained through transition state theory. The outcomes of this methodological innovation are captured in Equation (1).

(1) $$\log{\left(\frac{k}{k_{H}}\right)}_{\mathrm{ideal}}=\;8.61\;\left(q_{2}+q_{5}\right)-76.00\;F+53.25\;\mathrm{with}\;F=log\left\{{\;\chi }_{M}\;[S+\alpha \left(s_{2}^{+}+s_{5}^{+}\right)]+\beta /(s_{2}^{+}+s_{5}^{+})\right\}$$

Herein, k and $k_{H}$ are the rate constants for the modified and unmodified thiophen determined using the ωB97X-D3/def2-TZVP level of theory, in gas phase. $q_{2}$ and $q_{5}$ represent the Hirshfeld charges (in electron unit) on carbon atoms 2 and 5 within the thiophene moieties, respectively. Analogously, $s_{2}^{+}$ and $s_{5}^{+}$ describe the condensed local softness (in eV^−1^·electron unit) for nucleophilic attacks (singlet oxygen) targeting carbon atoms 2 and 5. The parameters S and${\;\chi }_{M}$ refer to the global chemical softness (in eV^−1^ unit) and Mulliken electronegativity (in eV unit) of the compound, in that order. The Greek letters α and β are constants that fine-tune the fit and ensure an appropriate unit conversion, with α defined as 30.75 (eV^2^·electron)^−1^ and β equating to 0.03822 eV^−1^·electron. Finally, the numerical data associated with Equation (1) can be found in Table S1.

**Figure 3:**
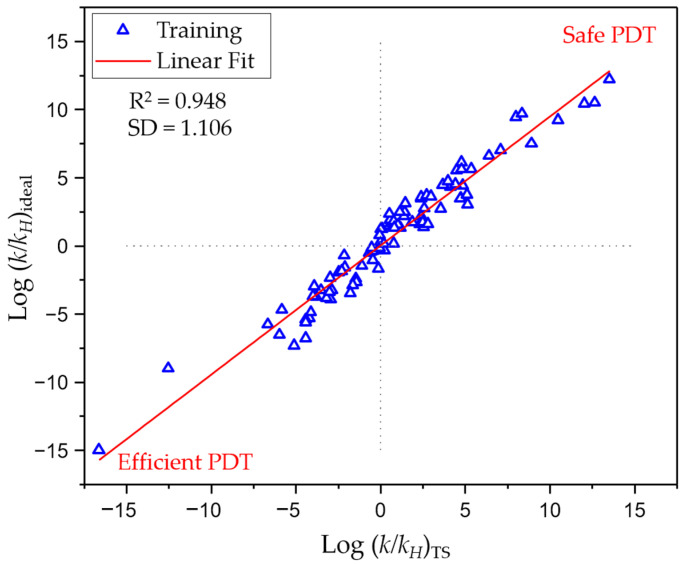
Plot of log ($k/k_{H}$)_TS_ vs. log ($k/k_{H}$)_ideal_ for singlet-oxygen oxidation of thiophenes. The log ($k/k_{H}$)_TS_ values are based on transition state theory, computed in a vacuum using the ωB97X-D3/def2-TZVP level of theory and Equation (1). The log ($k/k_{H}$)_ideal_ values are derived from conceptual DFT applying Equation (2) solely to the reactant. Triangle markers represent training data points, and the red line indicates the linear fit.

In contrast to the correlation depicted in Figure 2A, Equation (1) incorporates three additional descriptors into the reactivity estimation: the Hirshfeld charge ($\chi_{M}$), global softness (S), and condensed softness ($s_{2}^{+}$ and $s_{5}^{+}$). The incorporation of these indices into the model markedly improves the predictive power regarding the kinetic behavior of the thiophens. This improvement is evidenced by the increase in the coefficient of determination from R^2^ = 0.759 to R^2^ = 0.949 when comparing results in Figure 2A with those in Figure 3. This enables the estimation of the reactivity of thiophene-containing photosensitizers through Equation (1), based solely on the reactant structure, thereby circumventing the necessity for TS characterization. The precision of this model is highlighted by a standard deviation (SD) of 1.096 which, when presented on a logarithmic scale, denotes an order of magnitude in the context of relative rate constants. This degree of precision indicates the capacity of the model to discern between the reactivities of two photosensitizers, even when their estimated reactivity differs by two orders of magnitude.

While there is significant variability in discriminating thiophenes with similar reactivities, precision in this regard is not necessary in PDT. In PDT, the primary goal is to evaluate the reactivity of photosensitizers, categorizing them as either safe or efficient therapeutic agents based on their resistance to singlet oxygen oxidation. In this sense, Equation (1) represents a significant advancement in modeling the reactivity of thiophene-containing photosensitizers, offering a streamlined and computationally inexpensive alternative to conventional approaches.

### 2.4. Conceptual Interpretation of the Model

A closer examination of Equation (1) reveals that it incorporates the cumulative charges ($q_{2}$ + $q_{5}$) of the alpha carbon atoms to sulfur, along with an F term associated with Mulliken electronegativity, local softness, and global softness. A positive coefficient of 8.61 for ($q_{2}$ + $q_{5}$) suggests that generating positive charges on carbon atoms 2 and 5 accelerates the oxidation reaction, which is reasonable. However, this correlation is not the predominant driving force in thiophene reactivity. The inclusion of these charges in the model improves the fit only by 2.1%, in stark contrast to the overarching influence of the F term, which contributes 97.9% to the R^2^ value. This finding corroborates the non-ionic character often associated with pericyclic reactions.

The F term introduces an interesting interplay among descriptors, with a non-linear expression with respect to “$s_{2}^{+}+s_{5}^{+}$” in the logarithmic operation. This expression, when contrasted with the expression in Figure 2, represents a nuanced variation of $\chi_{M}$.

As with log ($\chi_{M}$), illustrated in Figure 2, the F term in Equation (1) exhibits a negative correlation with the rate constant of thiophenes, indicated by its negative coefficient (−76.00). This implies that a higher F value corresponds to a decreased reactivity of thiophenes. In this context, all factors included in the logarithmic expression of F serve to reduce the reactivity as they increase, given their positive coefficients (α = 30.75 and β = 0.03822). Keeping this in mind, the term β/$\left(s_{2}^{+}+s_{5}^{+}\right)$ becomes larger as $\left(s_{2}^{+}+s_{5}^{+}\right)$ decreases, indicating that reactivity diminishes when $\left(s_{2}^{+}+s_{5}^{+}\right)$ increases. This is reasonable since lower local condensed softness in the alpha atoms to sulfur correlates with reduced reactivity toward singlet oxygen attack. On the other hand, the expression ${\;\chi }_{M}\;[S+\alpha \left(s_{2}^{+}+s_{5}^{+}\right)]$ consists of two components with opposing responses to reactivity: an increase in $\chi_{M}\;$leads to a decrease in reactivity, whereas an increase in $\left[S+\alpha \left(s_{2}^{+}+s_{5}^{+}\right)\right]$ enhances it. This suggests that $\left[S+\alpha \left(s_{2}^{+}+s_{5}^{+}\right)\right]$ balances out the impact of electronegativity alterations resulting from substituent modifications. Concurrently, $\chi_{M}\;$mitigates the reactivity linked with the softness parameter, particularly in the context of singlet oxygen attack.

### 2.5. Fitting the Model to the Experimental Data

The model described in Equation (1) presents an idealized depiction of the kinetic behavior of 90 thiophenes when exposed to singlet oxygen in a vacuum, without solvent effect. This model is grounded in a theoretical framework conducted at an appropriate level of theory. Consequently, it offers a conceptual and idealized perspective on the reactivity of thiophenes toward oxidation by singlet oxygen.

(2) $$\log{\left(\frac{k}{k_{2T}}\right)}_{Methanol}=\;0.525\log{\left(\frac{k}{k_{H}}\right)}_{ideal}+8.646$$

**Figure 4:**
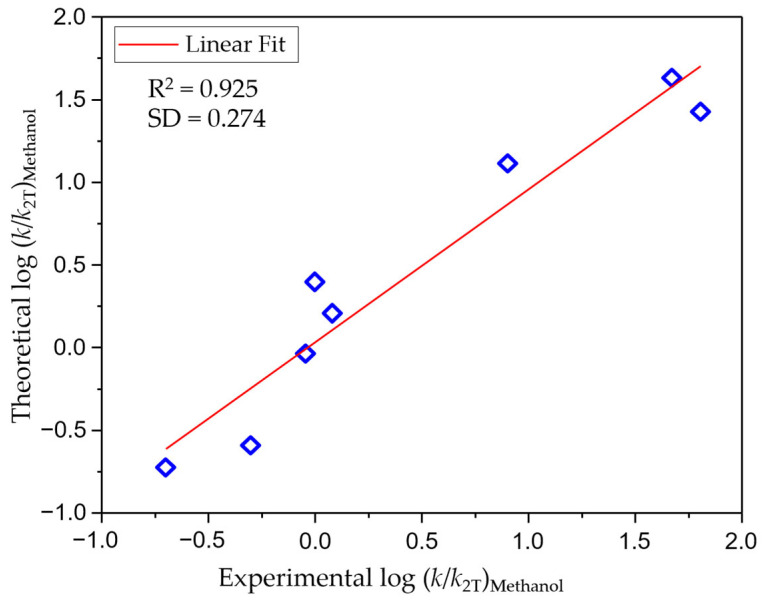
Plot of experimental log ($k/k_{2T}$)_Methanol_ vs. theoretical log ($k/k_{2T}$)_Methanol_ for singlet-oxygen oxidation of thiophenes in methanol. The theoretical log ($k/k_{2T}$)_Methanol_ is calculated according to Equation (3), with *β* in log ($k/k_{H}$)_ideal_ (referenced in Equation (1)) equal to 0.11686. The diamond markers indicate the specific points of convergence between the experimental and theoretical values.

In the context of this idealized framework, as delineated in Equation (1), it becomes feasible to approximate the experimental findings reported in the literature through simple adjustments. Table 2 displays the juxtaposition of both experimentally derived and theoretically estimated relative rate constants for a set of eight thiophenes, relative to 2-tert-butylthiophene (2T), using a logarithmic function (log($k/k_{2T}$)_Methanol_). It is important to note that the empirical rate constants, determined in methanol by van Tilborg [52], do not include the unmodified thiophene. This omission renders the conversion of $k/k_{2T}$ into $k/k_{H}$ infeasible. Nonetheless, the experimentally observed rate constants relative to 2T were successfully predicted by employing Equation (2) (adjusted ideal model), demonstrating a significant correlation of 92.5%, as illustrated in Figure 4. The numerical data associated with Equation (2) can be found in Table S2.

This implies that the development of a conceptual model (Equation (1)) based on transition state theory, combined with quantum mechanical calculations, conceptual DFT, and genetic programming, captures the kinetic behavior of thiophenes in the presence of singlet oxygen. Furthermore, its proper adjustment has the potential to estimate their reactivity under real conditions, as demonstrated with Equation (2).

### 2.6. Evaluating the Reactivity of Some Thiophene-Based Photosensitizers

**Scheme 4:**
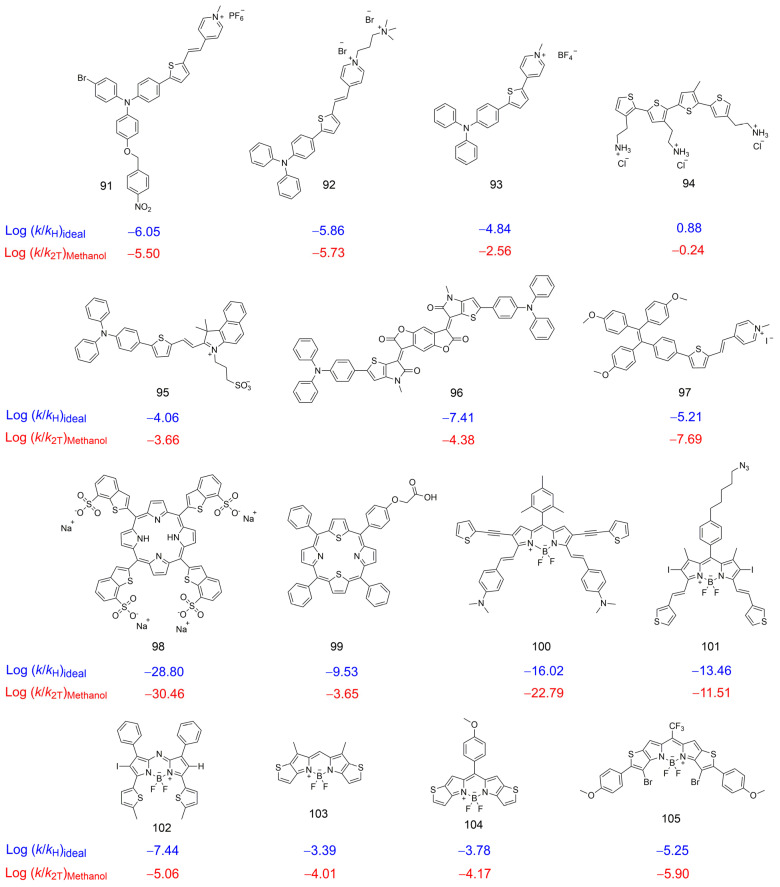
Reactivity for some photosensitizers as predicted by Equation (1) (in blue) and Equation (2) (in red). Log ($k/k_{H}$)_ideal_ represents the reactivity excluding solvent effects, measured relative to the rate constant of unmodified thiophene (*k*_H_). Log ($k/k_{2T}$)_Methanol_ denotes the reactivity in a methanolic environment, compared against the rate constant of 2-tert-butylthiophene ($k_{2T}$). The photosensitizers framed herein are derived from the literature [14,20,21,22,24,27,28,39,43,45,46,48,73,74].

Scheme 4 presents a range of compounds, **91**–**105**, with distinct properties and applications in the field of PDT [14,20,21,22,24,27,28,39,43,45,46,48,73,74]. Compounds **91**–**97** showcase Aggregation-Induced Emission (AIE) characteristics, a novel attribute enhancing luminescence and photodynamic activity in aggregated states, making them suitable for targeted applications [75]. Meanwhile, compounds **98** and **99** are part of the esteemed porphyrinoid family, a group highly recognized for its pivotal role in PDT research. Compounds **100**–**105**, belonging to the bodipy family, provide an alternative to porphyrinic compounds, broadening the scope of options in this field. A common feature among all these compounds is the presence of at least one thiophene moiety in their structure.

The results indicate that, with the exception of compound **94**, all photosensitizers exhibit reactivity at least three orders of magnitude lower than unmodified thiophene, rendering them 1000 times more stable. This discovery is crucial as it suggests that photosensitizers based on thiophene with reactivities of log ($k/k_{H}$)_ideal_ ≤ −3 could demonstrate effective photodynamic action, countering the potential mechanisms of singlet oxygen oxidation. This is particularly exemplified by compound **103** which, with reactivities of log ($k/k_{H}$)_ideal_ = −3.39, exhibits a singlet oxygen quantum yield (Φ_Δ_) of 58.1% and a triplet quantum yield (Φ_T_) of 63.7% [45]. This indicates that for compound **103**, processes such as radiative and non-radiative deactivations of its triplet state, or chemical quenching by singlet oxygen, are relatively insignificant. Similarly, the estimated reactivity in methanol indicates that photodynamic action is maintained effectively when log ($k/k_{2T}$)_Methanol_ ≤ −4. Conversely, photosensitizers with log ($k/k_{H}$)_ideal_ > −3 o log ($k/k_{2T}$)_Methanol_ > −4 could be categorized within the safer spectrum of PDT agents, albeit at the expense of their photosensitizing efficiency.

### 2.7. Script-Like Tool Description

To facilitate the application of our methodology, we have developed a script that automates the calculation of reactivities using only the output or single points from the N, N − 1, and N + 1 electron states of thiophene-containing compounds. The script is available at the following link: https://github.com/Jacksonalcazar/Thiophene-Reactivity-toward-Singlet-Oxygen (accessed on 19 December 2023).

## 3. Materials and Methods

### 3.1. Choice of Theoretical Level

The theoretical level was selected based on the reproduction of experimental data previously reported by van Tilborg [52]. The trialed methods included B3LYP-D3BJ/def2-TZVP [76,77,78,79], M06-2X/def2-TZVP [79,80], M06-2X-D3(0)/def2-TZVP [79,80,81], ωB97X-D3/TZVP [82], ωB97X-D3/def2-TZVP [79,82], and ωB97X-D3/ma-def2-TZVP [82,83]. These levels of theories are highly recommended because of their robust performance in predicting barrier heights and thermodynamic data across a broad spectrum of over 200 chemical reactions [67]. Additionally, the B97-3C method was examined given its commendable balance between reliability and computational cost [84].

The process was initiated with the geometric optimization of the compounds 2,5-dimethylthiophene, 2,5-di-tert-butylthiophene, 2,4-di-tert-butylthiophene, 2-methylthiophene, 2-tert-butylthiophene, 2,5-diphenylthiophene, 3-methylthiophene, and 2-bromothiophene, along with their respective endoperoxide products, as depicted in Scheme 1. These optimizations were conducted in the presence of an implicit methanol solvent modeled using the conductor-like polarizable continuum model with the COSMO epsilon function (CPCMC) [85,86].

Transition state (TS) identification was accomplished utilizing the Nudged Elastic Band with TS optimization method (NEB-TS) based on the optimized geometries of the reactants and product [87]. Vibrational mode analysis and optimization convergence were ensured for isolated reactants, products, and TSs. All reactants and products displayed exclusively positive frequencies, whereas every TS exhibited a singular imaginary frequency. An IRC analysis was employed to ensure the located TS connected the desired reactants and products [88]. Upon careful verification of all outputs, the Gibbs free energy was extracted and subsequently employed to compute the rate constant utilizing the Eyring’s Transition State Theory equation (Equation (3)) [61,62]:

(3) $$k=\gamma \frac{k_{B}T}{h}e^{-\frac{{∆G}^{‡}}{RT}}$$

Herein, the rate constant (k) is dictated by the Boltzmann constant, $k_{B}$, temperature, T, Planck’s constant, h, the universal gas constant, R, the Gibbs free energy of activation, ${∆G}^{‡}$, and the transmission coefficient, γ, which was uniformly considered as 1 across all computations.

This methodology was systematically applied with each of the previously mentioned DFT levels of theory. The results were then compared with experimental data to evaluate the performance employing the square of the Pearson correlation coefficient (r^2^).

All geometric optimizations and frequency calculations were performed using the ORCA software package (Program Version 5.0.3) [89,90]. We employed the auxiliary basis set def2/J and utilized the RIJCOSX approximation to expedite hybrid DFT calculations [91,92]. To ensure a high level of precision in the computed properties, the defgrid3 grid level was chosen for our DFT calculations [90]. All computations adhered to the TightSCF convergence protocol.

### 3.2. Kinetics of Singlet-Oxygen Oxidation of Thiophene Derivatives

A series of thiophene derivatives were designed to assess the influence of substituents and of the structural expansion of the thiophene scaffold on the rate constant for oxidation by singlet oxygen. The structural modifications are illustrated in Scheme 2. All electronic effects, including those related to the expansion of the thiophene ring, were covered using diverse substituents; this resulted in a total of 90 derivatives.

For each thiophene derivative, the determination of the TS was achieved using the NEB-TS method in a vacuum to eliminate solvent effects [87]. The identified transition states were further verified using IRC calculations. For our calculations, we employed the ωB97X-D3/def2-TZVP level of theory [79,82], in conjunction with the auxiliary basis set def2/J. To speed up hybrid DFT calculations, we used the RIJCOSX approximation with the TightSCF convergence protocol and the defgrid3 grid level [91,92], as implemented in the ORCA program (Version 5.0.3) [89,90]. Notably, the ωB97X-D3/def2-TZVP method/basis set exhibited the highest Pearson correlation with experimental data (see Table 1).

The rate constant for each derivative was calculated using Equation (1) and then normalized to the rate constant of the unmodified thiophene.

### 3.3. Development of a Reactivity Prediction Model Using Conceptual DFT

In the realm of quantum chemistry, understanding and predicting molecular reactivity is paramount for numerous applications, ranging from drug design to material science [93,94,95,96,97]. Density Functional Theory offers a computationally efficient approach to addressing these challenges. Specifically, Conceptual DFT (CDFT) [63,98], a subfield that focuses on linking DFT quantities to chemical reactivity, has emerged as a powerful tool [99,100,101,102,103]. In this methodology, we detail the development of a model for predicting the reactivity of thiophene derivatives based on their structure and the conceptual DFT principles involved.

#### 3.3.1. Conceptual DFT Quantities:

Central to any study involving CDFT is the requirement of information regarding electronic energy (E) and electron density for states with N, N + 1, and N − 1 electrons. Typically, N represents the number of electrons that a system possesses in its most stable configuration. For all calculations, including those involving states with N + 1 and N − 1 electrons, it is imperative to use the geometry optimized for the N electron.

Several global indices help understand and analyze the reactivity of molecules. Vertical Ionization Potential (VIP) is the energy difference between states N and N − 1. Vertical Electron Affinity (VEA) is the energy difference between states N and N + 1. Mulliken Electronegativity ($\chi_{M}$) is the arithmetic average of VIP and VEA [104,105]. Chemical Potential is the negative of $\chi_{M}$ [105]. Hardness is the difference between VIP and VEA, and can also be equated to the fundamental gap [106]. Softness is the reciprocal of hardness [107]. Electrophilicity Index [108] and Nucleophilicity Index [109] are parameters reflecting the electrophilic and nucleophilic tendencies of molecules, respectively.

Expanding on the theme of molecular reactivity, real space functions come into play. The Fukui function [110] and local softness [107,111] offer critical insights into the loci of nucleophilic, electrophilic, and radical attacks. Similarly, atom indices encompass condensed Fukui functions [112,113] and local softness [112,113] metrics that provide insights for various types of chemical attacks, such as nucleophilic, electrophilic, and radical.

#### 3.3.2. Model Construction

To develop the model, all descriptors based on conceptual DFT were computed using the ORCA software (version 5.0.3). The local descriptors related to atomic charge, such as condensed Fukui functions, were derived from the Hirshfeld charge [113] on the carbon atoms of the thiophene scaffold directly involved in the singlet oxygen cycloaddition (at the 2′ and 5′-carbon positions).

In this study, a total of 90 thiophene derivatives were evaluated. Electronic descriptors for these compounds were calculated and then subjected to symbolic regression via the genetic programming learning method, utilizing the Python library [59,60]. The dependent variable in this analysis was the logarithm of the relative rate constant ratio, denoted as log ($k/k_{H}$)_ideal_, where k and $k_{H}$ represent the modified and unmodified thiophenes, respectively.

To refine the genetic programming approach, several key parameters were adjusted. The population size was set at 5000, and the generation count was established at 10,000. The probabilities for crossover and subtree mutation were determined to be 0.7 and 0.1, respectively. The maximum sample size was capped at 0.9, and the parsimony coefficient was fixed at 0.01. The symbolic regression encompassed a comprehensive range of operations, including addition, subtraction, multiplication, division, square root, logarithm, and inversion. Throughout the process, the determination coefficient (R^2^) served as the primary metric for monitoring evolutionary progress.

## 4. Conclusions

This study presents a groundbreaking mathematical model that harnesses CDFT and genetic programming to predict the reactivity of thiophene toward singlet oxygen in PDT. Our research bridges advanced computational methods, including various DFT techniques and symbolic regression, with experimental validation to deliver a robust tool for aiding in the design of thiophene-based photosensitizing agents. The model demonstrates a remarkable ability to classify thiophene-containing photosensitizers based on their photodynamic efficiency and safety, revealing that photosensitizers incorporating thiophene with significantly lower reactivity than unmodified thiophene retain their photodynamic performance without substantial singlet oxygen quenching.

Key insights into the impact of electronic effects on thiophene reactivity offer a deeper understanding of the molecular underpinnings of photosensitizer design. By achieving a delicate balance between efficiency and safety, our model provides a pathway to the development of therapeutic agents that harness the full potential of thiophene-based photosensitizers. The integration of CDFT with genetic programming not only elucidates the reactivity trends of thiophenes but also paves the way for their rational design, ensuring optimal therapeutic outcomes.

Furthermore, the development of a user-friendly script enhances the accessibility of our model, allowing for its massification and facilitating the modulation of efficiency and safety in the design of PDT agents containing thiophene moieties. This study marks a significant step forward in the quest for optimal photosensitizer design, offering a promising direction for future research in the field of PDT.

## Figures and Tables

**Table 1:** DFT levels of theory and their corresponding Pearson Correlation Coefficient Squared (r^2^) for the relationship between experimental ^a^ and theoretical rate constants in the oxidation of thiophenes by singlet oxygen.

Level of Theory	r^2^
B97-3C	0.8184
B3LYP-D3BJ/def2-TZVP	0.9437
M06-2X/def2-TZVP	0.5021
M06-2X-D3(0)/def2-TZVP	0.7573
ωB97X-D3/TZVP	0.9420
ωB97X-D3/def2-TZVP	0.9838
ωB97X-D3/ma-def2-TZVP	0.9109

^a^ Adapted with permission from Ref. [52]. Copyright © 2024, WILEY-VCH Verlag GmbH & Co. KGaA, Weinheim, Germany.

**Table 2:** Logarithm of the Experimental and Theoretical Relative Rate Constants.

ID	$\mathrm{Log}\;(k/k_{2T}$)_Methanol_^a,b^ Experimental	$\mathrm{Log}\;(k/k_{2T}$)_Methanol_^a,c^ Theoretical
2	0.08	0.21
3	0.00	0.40
8	−0.70	−0.73
21	−0.30	−0.59
40	1.81	1.43
42	1.67	1.63
54	−0.05	−0.04
59	0.90	1.11

^a^ *k*_2T_ represents the oxidation rate constant of 2-tert-butylthiophene, and the subscript ‘Methanol’ denotes the methanolic phase. ^b^ Reprinted with permission from Ref. [52]. Copyright © 2024, WILEY-VCH Verlag GmbH & Co. KGaA, Weinheim, Germany. ^c^ Calculated according to Equation (2), with β in log ($k/k_{H}$)_ideal_ (referenced in Equation (1)) equal to 0.11686.

## Data Availability

The data files for this manuscript, which include inputs, outputs, and optimized structures, are accessible via the following link: https://zenodo.org/records/10530896 (accessed on 18 January 2024). Additionally, the source code and prediction model script associated with this manuscript can be found at: https://github.com/Jacksonalcazar/Thiophene-Reactivity-toward-Singlet-Oxygen (accessed on 19 December 2023).

## Supplementary Materials

The following supporting information can be downloaded at: https://www.mdpi.com/article/10.3390/ijms25052528/s1.

## References

[1] Gunaydin G., Gedik M.E., Ayan S. (2021). Photodynamic Therapy for the Treatment and Diagnosis of Cancer–A Review of the Current Clinical Status. Front. Chem..

[2] Juarranz Á., Gilaberte Y., González S. (2020). Photodynamic therapy (PDT) in oncology. Cancers.

[3] Shi H., Sadler P.J. (2020). How promising is phototherapy for cancer?. Br. J. Cancer.

[4] Kessel D. (2023). Photodynamic Therapy: Critical PDT Theory. Photochem. Photobiol..

[5] Sharma D., Singh S., Kumar P., Jain G.K., Aggarwal G., Almalki W.H., Kesharwani P. (2023). Mechanisms of photodynamic therapy. Nanomaterials for Photodynamic Therapy.

[6] Correia J.H., Rodrigues J.A., Pimenta S., Dong T., Yang Z. (2021). Photodynamic therapy review: Principles, photosensitizers, applications, and future directions. Pharmaceutics.

[7] Sai D.L., Lee J., Nguyen D.L., Kim Y.P. (2021). Tailoring photosensitive ROS for advanced photodynamic therapy. Exp. Mol. Med..

[8] Maharjan P.S., Bhattarai H.K. (2022). Singlet Oxygen, Photodynamic Therapy, and Mechanisms of Cancer Cell Death. J. Oncol..

[9] Pham T.C., Nguyen V.N., Choi Y., Lee S., Yoon J. (2021). Recent Strategies to Develop Innovative Photosensitizers for Enhanced Photodynamic Therapy. Chem. Rev..

[10] Tavakkoli Yaraki M., Liu B., Tan Y.N. (2022). Emerging Strategies in Enhancing Singlet Oxygen Generation of Nano-Photosensitizers Toward Advanced Phototherapy. Nano-Micro Lett..

[11] Pang E., Zhao S., Wang B., Niu G., Song X., Lan M. (2022). Strategies to construct efficient singlet oxygen-generating photosensitizers. Coord. Chem. Rev..

[12] Cakmak Y., Kolemen S., Duman S., Dede Y., Dolen Y., Kilic B., Kostereli Z., Yildirim L.T., Dogan A.L., Guc D. (2011). Designing excited states: Theory-guided access to efficient photosensitizers for photodynamic action. Angew. Chem. Int. Ed..

[13] Gao J., Yang H., Lu Y., Shi Q., Xu S., Wu W., Hu F., Liu B. (2023). Anthracene-Bridged Photosensitizers for Effective and Safe Photodynamic Therapy. Chem. Mater..

[14] Shi J., Wang Z., Shen C., Pan T., Xie L., Xie M., Huang L., Jiang Y., Zhou J., Zuo W. (2022). Hypoxia degradable AIE photosensitizer with high efficiency of photodynamic therapy and improved biological safety. Dye. Pigment..

[15] Huang H., Xie W., Wan Q., Mao L., Hu D., Sun H., Zhang X., Wei Y. (2022). A Self-Degradable Conjugated Polymer for Photodynamic Therapy with Reliable Postoperative Safety. Adv. Sci..

[16] Yuan B., Wu H., Wang H., Tang B., Xu J., Zhang X. (2021). A Self-Degradable Supramolecular Photosensitizer with High Photodynamic Therapeutic Efficiency and Improved Safety. Angew. Chem..

[17] Akimoto J., Fukami S., Kohno M. (2023). Efficacy and safety of photodynamic therapy using talaporfin sodium and a semiconductor laser in patients with malignant meningeal tumors. Photodiagnosis Photodyn. Ther..

[18] Li H., Long G., Tian J. (2023). Efficacy and safety of photodynamic therapy for non–muscle-invasive bladder cancer: A systematic review and meta-analysis. Front. Oncol..

[19] Tan G., Xu J., Yu Q., Yang Z., Zhang H. (2022). The safety and efficiency of photodynamic therapy for the treatment of osteosarcoma: A systematic review of in vitro experiment and animal model reports. Photodiagnosis Photodyn. Ther..

[20] Liu Q., Tian J., Tian Y., Sun Q., Sun D., Liu D., Wang F., Xu H., Ying G., Wang J. (2021). Thiophene donor for NIR-II fluorescence imaging-guided photothermal/photodynamic/chemo combination therapy. Acta Biomater..

[21] Wang D., Su H., Kwok R.T.K., Hu X., Zou H., Luo Q., Lee M.M.S., Xu W., Lam J.W.Y., Tang B.Z. (2018). Rational design of a water-soluble NIR AIEgen, and its application in ultrafast wash-free cellular imaging and photodynamic cancer cell ablation. Chem. Sci..

[22] Khatoon S.S., Chen Y., Zhao H., Lv F., Liu L., Wang S. (2020). In situ self-assembly of conjugated polyelectrolytes for cancer targeted imaging and photodynamic therapy. Biomater. Sci..

[23] Zhuang W., Yang L., Ma B., Kong Q., Li G., Wang Y., Tang B.Z. (2019). Multifunctional Two-Photon AIE Luminogens for Highly Mitochondria-Specific Bioimaging and Efficient Photodynamic Therapy. ACS Appl. Mater. Interfaces.

[24] Zhou Z., Ergene C., Lee J.Y., Shirley D.J., Carone B.R., Caputo G.A., Palermo E.F. (2019). Sequence and Dispersity Are Determinants of Photodynamic Antibacterial Activity Exerted by Peptidomimetic Oligo(thiophene)s. ACS Appl. Mater. Interfaces.

[25] Huang J., He B., Zhang Z., Li Y., Kang M., Wang Y., Li K., Wang D., Tang B.Z. (2020). Aggregation-Induced Emission Luminogens Married to 2D Black Phosphorus Nanosheets for Highly Efficient Multimodal Theranostics. Adv. Mater..

[26] He Z., Zhao L., Zhang Q., Chang M., Li C., Zhang H., Lu Y., Chen Y. (2020). An Acceptor–Donor–Acceptor Structured Small Molecule for Effective NIR Triggered Dual Phototherapy of Cancer. Adv. Funct. Mater..

[27] Zhu W., Kang M., Wu Q., Zhang Z., Wu Y., Li C., Li K., Wang L., Wang D., Tang B.Z. (2021). Zwitterionic AIEgens: Rational Molecular Design for NIR-II Fluorescence Imaging-Guided Synergistic Phototherapy. Adv. Funct. Mater..

[28] Chen J., Wen K., Chen H., Jiang S., Wu X., Lv L., Peng A., Zhang S., Huang H. (2020). Achieving High-Performance Photothermal and Photodynamic Effects upon Combining D–A Structure and Nonplanar Conformation. Small.

[29] Xu Y., Zhang Y., Li J., An J., Li C., Bai S., Sharma A., Deng G., Kim J.S., Sun Y. (2020). NIR-II emissive multifunctional AIEgen with single laser-activated synergistic photodynamic/photothermal therapy of cancers and pathogens. Biomaterials.

[30] Awuah S.G., Polreis J., Biradar V., You Y. (2011). Singlet oxygen generation by novel NIR BODIPY dyes. Org. Lett..

[31] Tanaka K., Yamane H., Yoshii R., Chujo Y. (2013). Efficient light absorbers based on thiophene-fused boron dipyrromethene (BODIPY) dyes. Bioorganic Med. Chem..

[32] Yang Y., Guo Q., Chen H., Zhou Z., Guo Z., Shen Z. (2013). Thienopyrrole-expanded BODIPY as a potential NIR photosensitizer for photodynamic therapy. Chem. Commun..

[33] Chen J., Burghart A., Derecskei-Kovacs A., Burgess K. (2000). 4,4-Difluoro-4-bora-3a,4a-diaza-s-indacene (BODIPY) dyes modified for extended conjugation and restricted bond rotations. J. Org. Chem..

[34] Dingyuan Y., Tan Y., Sun Y., Huang W., Zhu D., Yan D., Wang D., Tang B.Z. (2023). Thiophene π-bridge based NIR-II AIEgens for biomedical applications. Luminescence.

[35] Liu Z., Wang Q., Qiu W., Lyu Y., Zhu Z., Zhao X., Zhu W.H. (2022). AIE-active luminogens as highly efficient free-radical ROS photogenerator for image-guided photodynamic therapy. Chem. Sci..

[36] Dong M.J., Li W., Xiang Q., Tan Y., Xing X., Wu C., Dong H., Zhang X. (2022). Engineering Metal-Organic Framework Hybrid AIEgens with Tumor-Activated Accumulation and Emission for the Image-Guided GSH Depletion ROS Therapy. ACS Appl. Mater. Interfaces.

[37] Satyanarayan M.N., Trivedi D.R., Mohan M., Pangannaya S. (2022). Aggregation-induced emission in thiophene derivatives. ISSS J. Micro Smart Syst..

[38] Zhang Z., Xu W., Kang M., Wen H., Guo H., Zhang P., Xi L., Li K., Wang L., Wang D. (2020). An All-Round Athlete on the Track of Phototheranostics: Subtly Regulating the Balance between Radiative and Nonradiative Decays for Multimodal Imaging-Guided Synergistic Therapy. Adv. Mater..

[39] Xiong W., Wang L., Chen X., Tang H., Cao D., Zhang G., Chen W. (2020). Pyridinium-substituted tetraphenylethylene salt-based photosensitizers by varying counter anions: A highly efficient photodynamic therapy for cancer cell ablation and bacterial inactivation. J. Mater. Chem. B.

[40] Zhao N., Li P., Zhuang J., Liu Y., Xiao Y., Qin R., Li N. (2019). Aggregation-Induced Emission Luminogens with the Capability of Wide Color Tuning, Mitochondrial and Bacterial Imaging, and Photodynamic Anticancer and Antibacterial Therapy. ACS Appl. Mater. Interfaces.

[41] Xu W., Lee M.M.S., Zhang Z., Sung H.H.Y., Williams I.D., Kwok R.T.K., Lam J.W.Y., Wang D., Tang B.Z. (2019). Facile synthesis of AIEgens with wide color tunability for cellular imaging and therapy. Chem. Sci..

[42] Wang D., Lee M.M.S., Xu W., Kwok R.T.K., Lam J.W.Y., Tang B.Z. (2018). Theranostics based on AIEgens. Theranostics.

[43] Yıldız Gül E., Erdem M., Kazan H.H., Tanrıverdi Eçik E. (2023). Thiophene BODIPY-substituted cyclotriphosphazene-derived photosensitizers for photodynamic therapy applications. New J. Chem..

[44] Cai Y., Liang P., Tang Q., Yang X., Si W., Huang W., Zhang Q., Dong X. (2017). Diketopyrrolopyrrole-Triphenylamine Organic Nanoparticles as Multifunctional Reagents for Photoacoustic Imaging-Guided Photodynamic/Photothermal Synergistic Tumor Therapy. ACS Nano.

[45] Ji S., Ge J., Escudero D., Wang Z., Zhao J., Jacquemin D. (2015). Molecular structure-intersystem crossing relationship of heavy-atom-free bodipy triplet photosensitizers. J. Org. Chem..

[46] Wang D., Su H., Kwok R.T.K., Shan G., Leung A.C.S., Lee M.M.S., Sung H.H.Y., Williams I.D., Lam J.W.Y., Tang B.Z. (2017). Facile Synthesis of Red/NIR AIE Luminogens with Simple Structures, Bright Emissions, and High Photostabilities, and Their Applications for Specific Imaging of Lipid Droplets and Image-Guided Photodynamic Therapy. Adv. Funct. Mater..

[47] Rangasamy S., Ju H., Um S., Oh D.C., Song J.M. (2015). Mitochondria and DNA Targeting of 5,10,15,20-Tetrakis(7-sulfonatobenzo[b]thiophene) Porphyrin-Induced Photodynamic Therapy via Intrinsic and Extrinsic Apoptotic Cell Death. J. Med. Chem..

[48] Qi S., Kwon N., Yim Y., Nguyen V.N., Yoon J. (2020). Fine-tuning the electronic structure of heavy-atom-free BODIPY photosensitizers for fluorescence imaging and mitochondria-targeted photodynamic therapy. Chem. Sci..

[49] DeRosa M.C., Crutchley R.J. (2002). Photosensitized singlet oxygen and its applications. Coord. Chem. Rev..

[50] Moan J. (1986). Effect of bleaching of porphyrin sensitizers during photodynamic therapy. Cancer Lett..

[51] Bonnett R., Martínez G. (2001). Photobleaching of sensitisers used in photodynamic therapy. Tetrahedron.

[52] Van Tilborg W.J.M. (1976). Singlet-oxygen oxidation of thiophenes. Recl. Trav. Chim. Pays-Bas.

[53] Shi Q., Wu J. (2021). Review on Sulfur Compounds in Petroleum and Its Products: State-of-the-Art and Perspectives. Energy Fuels.

[54] Betiha M.A., Rabie A.M., Ahmed H.S., Abdelrahman A.A., El-Shahat M.F. (2018). Oxidative desulfurization using graphene and its composites for fuel containing thiophene and its derivatives: An update review. Egypt. J. Pet..

[55] Pham T.C., Cho M., Nguyen V.N., Van Nguyen T.K., Kim G., Min S., Kim M.R., Yoon J., Lee S. (2023). Regulating ^1^O_2_ generation from heavy-atom-free triplet photosensitizers based on thiophene-fused BODIPY. Dye. Pigment..

[56] Zhao S., Yang K., Jiang L., Xiao J., Wang B., Zeng L., Song X., Lan M. (2021). Polythiophene-Based Carbon Dots for Imaging-Guided Photodynamic Therapy. ACS Appl. Nano Mater..

[57] Fuse S., Takizawa M., Sato S., Okazaki S., Nakamura H. (2019). Elucidating the mode of action for thiophene-based organic D-π-A sensitizers for use in photodynamic therapy. Bioorganic Med. Chem..

[58] Orio M., Pantazis D.A., Neese F. (2009). Density functional theory. Photosynth. Res..

[59] Koza J.R. (1994). Genetic programming as a means for programming computers by natural selection. Stat. Comput..

[60] O’Neill M., Poli R., Langdon W.B., McPhee N.F. (2009). A Field Guide to Genetic Programming. Genet. Program. Evolvable Mach..

[61] Eyeing H. (1935). The activated complex and the absolute rate of chemical reactions. Chem. Rev..

[62] Mortimer R.G., Eyring H. (1980). Elementary transition state theory of the Soret and Dufour effects. Proc. Natl. Acad. Sci. USA.

[63] Chakraborty D., Chattaraj P.K. (2021). Conceptual density functional theory based electronic structure principles. Chem. Sci..

[64] Jorner K., Brinck T., Norrby P.O., Buttar D. (2021). Machine learning meets mechanistic modelling for accurate prediction of experimental activation energies. Chem. Sci..

[65] Mhadeshwar A.B., Wang H., Vlachos D.G. (2003). Thermodynamic Consistency in Microkinetic Development of Surface Reaction Mechanisms. J. Phys. Chem. B.

[66] Durant J.L. (1996). Evaluation of transition state properties by density functional theory. Chem. Phys. Lett..

[67] Mardirossian N., Head-Gordon M. (2017). Thirty years of density functional theory in computational chemistry: An overview and extensive assessment of 200 density functionals. Mol. Phys..

[68] Li Y., Fang D.C. (2014). DFT calculations on kinetic data for some [4 + 2] reactions in solution. Phys. Chem. Chem. Phys..

[69] Song X., Fanelli M.G., Cook J.M., Bai F., Parish C.A. (2012). Mechanisms for the reaction of thiophene and methylthiophene with singlet and triplet molecular oxygen. J. Phys. Chem. A.

[70] Skold C.N., Schlessinger R.H. (1970). The reaction of singlet oxygen with a simple thiophene. Tetrahedron Lett..

[71] Taft R.W. (1957). Concerning the electron-withdrawing power and the electronegativity of groups. J. Chem. Phys..

[72] Alcázar J.J., Misad Saide A.C., Campodónico P.R. (2023). Reliable and accurate prediction of basic pKa values in nitrogen compounds: The pKa shift in supramolecular systems as a case study. J. Cheminform..

[73] You Y., Gibson S.L., Hilf R., Davies S.R., Oseroff A.R., Roy I., Ohulchanskyy T.Y., Bergey E.J., Detty M.R. (2003). Water soluble, core-modified porphyrins. 3. Synthesis, photophysical properties, and in vitro studies of photosensitization, uptake, and localization with carboxylic acid-substituted derivatives. J. Med. Chem..

[74] Yu Z., Zhou J., Ji X., Lin G., Xu S., Dong X., Zhao W. (2020). Discovery of a Monoiodo Aza-BODIPY Near-Infrared Photosensitizer: In vitro and in vivo Evaluation for Photodynamic Therapy. J. Med. Chem..

[75] Hong Y., Lam J.W.Y., Tang B.Z. (2011). Aggregation-induced emission. Chem. Soc. Rev..

[76] Grimme S., Ehrlich S., Goerigk L. (2011). Effect of the damping function in dispersion corrected density functional theory. J. Comput. Chem..

[77] Becke A.D. (1992). Density-functional thermochemistry. I. The effect of the exchange-only gradient correction. J. Chem. Phys..

[78] Stephens P.J., Devlin F.J., Chabalowski C.F., Frisch M.J. (1994). Ab Initio calculation of vibrational absorption and circular dichroism spectra using density functional force fields. J. Phys. Chem..

[79] Weigend F., Ahlrichs R. (2005). Balanced basis sets of split valence, triple zeta valence and quadruple zeta valence quality for H to Rn: Design and assessment of accuracy. Phys. Chem. Chem. Phys..

[80] Zhao Y., Truhlar D.G. (2008). The M06 suite of density functionals for main group thermochemistry, thermochemical kinetics, noncovalent interactions, excited states, and transition elements: Two new functionals and systematic testing of four M06 functionals and 12 other functionals. Theor. Chem. Acc..

[81] Grimme S., Antony J., Ehrlich S., Krieg H. (2010). A consistent and accurate ab initio parametrization of density functional dispersion correction (DFT-D) for the 94 elements H-Pu. J. Chem. Phys..

[82] Lin Y.S., De Li G., Mao S.P., Chai J. (2013). Da Long-range corrected hybrid density functionals with improved dispersion corrections. J. Chem. Theory Comput..

[83] Zheng J., Xu X., Truhlar D.G. (2011). Minimally augmented Karlsruhe basis sets. Theor. Chem. Acc..

[84] Brandenburg J.G., Bannwarth C., Hansen A., Grimme S. (2018). B97-3c: A revised low-cost variant of the B97-D density functional method. J. Chem. Phys..

[85] Garcia-Ratés M., Neese F. (2020). Effect of the Solute Cavity on the Solvation Energy and its Derivatives within the Framework of the Gaussian Charge Scheme. J. Comput. Chem..

[86] Sinnecker S., Rajendran A., Klamt A., Diedenhofen M., Neese F. (2006). Calculation of solvent shifts on electronic g-tensors with the conductor-like screening model (COSMO) and its self-consistent generalization to real solvents (direct COSMO-RS). J. Phys. Chem. A.

[87] Ásgeirsson V., Birgisson B.O., Bjornsson R., Becker U., Neese F., Riplinger C., Jónsson H. (2021). Nudged Elastic Band Method for Molecular Reactions Using Energy-Weighted Springs Combined with Eigenvector following. J. Chem. Theory Comput..

[88] Ishida K., Morokuma K., Komornicki A. (1976). The intrinsic reaction coordinate. An ab initio calculation for HNC→HCN and H^−^+CH_4_→CH_4_+H^−^. J. Chem. Phys..

[89] Neese F., Wennmohs F., Becker U., Riplinger C. (2020). The ORCA quantum chemistry program package. J. Chem. Phys..

[90] Neese F. (2022). Software update: The ORCA program system—Version 5.0. Wiley Interdiscip. Rev. Comput. Mol. Sci..

[91] Weigend F. (2006). Accurate Coulomb-fitting basis sets for H to Rn. Phys. Chem. Chem. Phys..

[92] Neese F., Wennmohs F., Hansen A., Becker U. (2009). Efficient, approximate and parallel Hartree-Fock and hybrid DFT calculations. A “chain-of-spheres” algorithm for the Hartree-Fock exchange. Chem. Phys..

[93] Hughes T.B., Flynn N., Le Dang N., Swamidass S.J. (2021). Modeling the Bioactivation and Subsequent Reactivity of Drugs. Chem. Res. Toxicol..

[94] Melen R.L. (2019). Frontiers in molecular p-block chemistry: From structure to reactivity. Science.

[95] Yu J., Su N.Q., Yang W. (2022). Describing Chemical Reactivity with Frontier Molecular Orbitalets. JACS Au.

[96] Ertl P., Gerebtzoff G., Lewis R., Muenkler H., Schneider N., Sirockin F., Stiefl N., Tosco P. (2022). Chemical Reactivity Prediction: Current Methods and Different Application Areas. Mol. Inform..

[97] Hughes T.B., Le Dang N., Miller G.P., Swamidass S.J. (2016). Modeling reactivity to biological macromolecules with a deep multitask network. ACS Cent. Sci..

[98] Geerlings P., De Proft F., Langenaeker W. (2003). Conceptual density functional theory. Chem. Rev..

[99] Suresh C.H., Remya G.S., Anjalikrishna P.K. (2022). Molecular electrostatic potential analysis: A powerful tool to interpret and predict chemical reactivity. Wiley Interdiscip. Rev. Comput. Mol. Sci..

[100] Barrera Y., Anderson J.S.M. (2023). Predicting reactivity with a general-purpose reactivity indicator. Chem. React. Approaches Appl..

[101] Srivastava R. (2021). Chemical reactivity theory (CRT) study of small drug-like biologically active molecules. J. Biomol. Struct. Dyn..

[102] Lee B., Yoo J., Kang K. (2020). Predicting the chemical reactivity of organic materials using a machine-learning approach. Chem. Sci..

[103] Ehresmann B., Martin B., Horn A.H.C., Clark T. (2003). Local molecular properties and their use in predicting reactivity. J. Mol. Model..

[104] Mulliken R.S. (1934). A new electroaffinity scale; Together with data on valence states and on valence ionization potentials and electron affinities. J. Chem. Phys..

[105] Parr R.G., Donnelly R.A., Levy M., Palke W.E. (1977). Electronegativity: The density functional viewpoint. J. Chem. Phys..

[106] Parr R.G., Pearson R.G. (1983). Absolute Hardness: Companion Parameter to Absolute Electronegativity. J. Am. Chem. Soc..

[107] Yang W., Parr R.G. (1985). Hardness, softness, and the fukui function in the electronic theory of metals and catalysis. Proc. Natl. Acad. Sci. USA.

[108] Parr R.G., Szentpály L.V., Liu S. (1999). Electrophilicity index. J. Am. Chem. Soc..

[109] Domingo L.R., Chamorro E., Pérez P. (2008). Understanding the reactivity of captodative ethylenes in polar cycloaddition reactions. A theoretical study. J. Org. Chem..

[110] Parr R.G., Yang W. (1984). Density Functional Approach to the Frontier-Electron Theory of Chemical Reactivity. J. Am. Chem. Soc..

[111] Roy R.K., Krishnamurti S., Geerlings P., Pal S. (1998). Local softness and hardness based reactivity descriptors for predicting intra- and intermolecular reactivity sequences: Carbonyl compounds. J. Phys. Chem. A.

[112] Oláh J., Van Alsenoy C., Sannigrahi A.B. (2002). Condensed Fukui functions derived from stockholder charges: Assessment of their performance as local reactivity descriptors. J. Phys. Chem. A.

[113] Wang B., Rong C., Chattaraj P.K., Liu S. (2019). A comparative study to predict regioselectivity, electrophilicity and nucleophilicity with Fukui function and Hirshfeld charge. Theor. Chem. Acc..

