# Supplementary material for: Thiophene Stability in Photodynamic Therapy: A Mathematical Model Approach
Source: Int J Mol Sci. 2024 Feb 21;25(5):2528. doi: 10.3390/ijms25052528 (PMC10931787; doi:10.3390/ijms25052528)
Supplement: Supplementary file 1 [file ijms-25-02528-s001.zip › ijms-2865051-supplementary.pdf]

Supporting Information (SI)

Thiophene Stability in Photodynamic Therapy: A Mathematical Model Approach

Jackson J. Alcázar

Centro de Química Médica, Facultad de Medicina Clínica Alemana, Universidad del Desarrollo, Santiago 7780272, Chile; jackson.alcazar@udd.cl

Table of Contents

Table S1. Descriptors and Reactivities of 90 Thiophene Derivatives Calculated Based on Equations (1) and (3)..1

Table S2. Descriptors and Reactivities of 90 Thiophene Derivatives Calculated Based on Equation (2). 3

Table S1. Descriptors and Reactivities of 90 Thiophene Derivatives Calculated Based on Equations (1) and (3).

ID	$q_2 + q_5$	χ_M	S	$s_2^+ + s_5^+$	F	Log $(k/k_H)_{ideal}$	Log $(k/k_H)_{TS}$
1	-0.1180	3.8527	0.0913	0.0253	0.6865	0.0605	0.0000
2	-0.0736	3.4369	0.0986	0.0235	0.6483	3.3446	2.4047
3	-0.0733	3.4292	0.0996	0.0232	0.6469	3.4530	2.4115
4	-0.0718	3.3986	0.1009	0.0233	0.6454	3.5814	2.7184
5	-0.0944	4.3614	0.0974	0.0231	0.7138	-1.8153	-2.1096
6	0.0177	3.6603	0.0954	0.0249	0.6708	2.4228	2.6027
7	-0.0467	3.7969	0.1003	0.0237	0.6778	1.3319	0.5181
8	-0.0644	3.8106	0.1018	0.0233	0.6773	1.2166	0.1051
9	-0.0810	4.2695	0.1044	0.0193	0.6954	-0.3018	0.2398
10	-0.0809	4.3626	0.1060	0.0188	0.7005	-0.6832	-2.1275
11	-0.0762	4.6871	0.1082	0.0167	0.7162	-1.8387	-2.4550
12	0.0324	4.9836	0.1099	0.0232	0.7594	-4.1858	-3.5214
13	-0.0370	2.8801	0.1030	0.0241	0.6039	7.0344	7.0951
14	-0.0432	2.5027	0.1093	0.0247	0.5708	9.4962	8.0032
15	-0.0044	3.0707	0.1005	0.0244	0.6212	6.0012	4.7984
16	-0.0050	3.1537	0.0984	0.0247	0.6287	5.4230	4.4939
17	-0.0445	4.6589	0.1042	0.0218	0.7292	-2.5500	-2.9630
18	-0.0746	3.7015	0.1155	0.0150	0.6707	1.6304	1.1771
19	-0.0230	5.2380	0.1098	0.0135	0.7468	-3.7036	-2.9462
20	-0.0763	3.7495	0.1136	0.0176	0.6652	2.0344	0.5770
21	-0.1279	3.4775	0.0966	0.0256	0.6597	2.0126	1.3799

22	-0.1282	3.4430	0.0970	0.0253	0.6554	2.3386	1.4246
23	-0.1268	3.4619	0.0985	0.0249	0.6556	2.3295	1.1563
24	-0.0940	4.4303	0.0922	0.0247	0.7257	-2.7159	-1.4554
25	-0.1288	3.8374	0.0953	0.0266	0.6936	-0.5719	-0.0727
26	-0.1119	3.8873	0.0982	0.0262	0.6968	-0.6723	-0.5695
27	-0.1090	3.8950	0.0997	0.0262	0.6977	-0.7147	-0.6433
28	-0.0899	4.1167	0.1003	0.0222	0.6940	-0.2686	-0.0284
29	-0.0887	4.2275	0.1021	0.0207	0.6963	-0.4299	-0.4565
30	-0.0873	4.5584	0.1029	0.0183	0.7095	-1.4267	-1.1052
31	-0.0796	4.8621	0.1058	0.0177	0.7260	-2.6102	-1.4053
32	-0.1539	3.0213	0.1035	0.0270	0.6272	4.2554	4.1446
33	-0.1521	2.8344	0.1090	0.0268	0.6098	5.5961	4.7787
34	-0.1455	3.2632	0.1031	0.0271	0.6500	2.5938	3.5562
35	-0.1419	3.3709	0.0999	0.0270	0.6579	2.0262	2.4136
36	-0.0771	4.5814	0.0998	0.0238	0.7334	-3.1523	-1.6306
37	-0.1169	3.6490	0.1102	0.0160	0.6613	1.9801	0.7579
38	-0.0768	5.0992	0.1062	0.0156	0.7354	-3.3044	-1.7866
39	-0.1125	3.6298	0.1065	0.0193	0.6552	2.4822	0.5229
40	-0.0274	3.6496	0.0943	0.0192	0.6522	3.4488	4.7428
41	-0.0265	3.6435	0.0959	0.0187	0.6521	3.4619	4.7108
42	-0.0233	3.1833	0.1065	0.0205	0.6243	5.6017	5.3592
43	-0.0783	5.1178	0.0971	0.0199	0.7442	-3.9826	-3.7926
44	0.1544	3.6695	0.0959	0.0235	0.6654	4.0077	4.8704
45	0.0214	3.9382	0.1057	0.0212	0.6800	1.7514	0.7707
46	-0.0154	3.9673	0.1085	0.0203	0.6803	1.4120	0.0253
47	-0.0522	5.0099	0.1093	0.0155	0.7325	-2.8697	-3.9056
48	-0.0452	5.6084	0.1100	0.0131	0.7628	-5.1135	-4.2072
49	-0.0418	5.9600	0.1143	0.0128	0.7792	-6.3316	-5.9434
50	0.0526	2.4822	0.1077	0.0217	0.5664	10.6562	12.6587
51	0.0540	2.2097	0.1170	0.0216	0.5436	12.4042	13.5248
52	0.1140	2.7250	0.1009	0.0228	0.5868	9.6321	8.3709
53	0.0189	5.4732	0.1128	0.0195	0.7677	-4.9358	-5.8102
54	-0.0312	3.6671	0.1299	0.0148	0.6748	1.6939	2.5429
55	0.0544	6.3384	0.1115	0.0111	0.8000	-7.0838	-5.0853
56	-0.0351	3.7455	0.1283	0.0158	0.6737	1.7444	1.0287
57	-0.0831	3.2741	0.1002	0.0227	0.6333	4.4022	3.6861
58	-0.0838	3.2699	0.1025	0.0221	0.6322	4.4825	4.4384
59	-0.0795	3.2399	0.1040	0.0220	0.6300	4.6868	4.0029
60	-0.0753	4.8904	0.0975	0.0228	0.7469	-4.1656	-3.1680
61	0.0041	3.8228	0.0953	0.0251	0.6846	1.2552	2.3531
62	-0.0445	4.0373	0.1033	0.0235	0.6956	0.0020	0.2092

63	-0.0601	4.0658	0.1058	0.0230	0.6961	-0.1723	-0.5058
64	-0.0560	4.5459	0.1073	0.0205	0.7176	-1.7691	-0.1106
65	-0.0570	4.6053	0.1085	0.0201	0.7199	-1.9529	-2.2813
66	-0.0543	5.0887	0.1083	0.0170	0.7372	-3.2478	-2.8548
67	-0.0449	5.5303	0.1104	0.0171	0.7601	-4.9045	-4.1015
68	-0.0743	2.4962	0.1060	0.0213	0.5675	9.4823	10.4807
69	-0.0733	2.3144	0.1121	0.0219	0.5518	10.6813	12.0389
70	-0.0384	2.7962	0.1053	0.0245	0.5977	7.4935	8.9236
71	-0.0312	2.9481	0.1007	0.0246	0.6108	6.5570	6.4025
72	-0.0105	5.3509	0.1071	0.0224	0.7755	-5.7789	-4.4144
73	-0.0743	3.6734	0.1217	0.0163	0.6659	2.0016	2.4958
74	0.0072	5.9576	0.1087	0.0149	0.7740	-5.5117	-4.4242
75	-0.0720	3.7214	0.1178	0.0166	0.6666	1.9671	1.8826
76	-0.0941	3.3649	0.1128	0.0182	0.6398	3.8178	2.9717
77	-0.1319	3.4310	0.1078	0.0205	0.6431	3.2379	1.4693
78	-0.0651	3.1826	0.1279	0.0146	0.6484	3.4095	5.1481
79	-0.1652	3.0840	0.1153	0.0261	0.6328	3.7331	5.1076
80	-0.0444	3.7489	0.1118	0.0152	0.6708	1.8879	2.7953
81	-0.1211	3.7858	0.1058	0.0168	0.6657	1.6095	0.8383
82	-0.0896	4.0057	0.1083	0.0180	0.6789	0.8815	-0.0388
83	-0.1148	4.0574	0.1053	0.0204	0.6854	0.1684	0.7939
84	-0.0654	4.2510	0.1198	0.0143	0.7033	-0.7629	-0.4531
85	-0.0266	4.4662	0.1665	0.0129	0.7384	-3.0983	-2.9895
86	0.1637	5.2855	0.1571	0.0174	0.7676	-3.6791	-3.9829
87	-0.0473	5.1767	0.1711	0.0116	0.7802	-6.4517	-4.4167
88	0.1510	7.1324	0.1435	0.0142	0.8344	-8.8620	-12.5334
89	-0.0083	3.7306	0.1188	0.0057	0.8921	-14.6223	-16.6258
90	-0.0457	5.0163	0.1924	0.0187	0.7703	-5.6903	-6.6432

Table S2. Descriptors and Reactivities of 90 Thiophene Derivatives Calculated Based on Equation (2).

ID	$q_2 + q_5$	χ_M	S	$s_2^+ + s_5^+$	F	Log $(k/k_H)_{ideal}$	Log $(k/k_{2T})_{Methanol}$ Calculated
2	-0.0814	3.4761	0.1736	0.0447	0.9029	-16.0709	0.0792
3	-0.0798	3.4741	0.1727	0.0430	0.8983	-15.7118	0.0000
8	-0.0700	3.7903	0.1785	0.0441	0.9276	-17.8495	-0.6990
21	-0.1372	3.5208	0.1690	0.0485	0.9166	-17.5942	-0.3010
40	-0.0347	3.2912	0.1785	0.0395	0.8776	-13.7497	1.8062
42	-0.0309	3.2857	0.1764	0.0360	0.8729	-13.3597	1.6721
54	-0.0437	3.7432	0.2285	0.0319	0.9133	-16.5384	-0.0458
59	-0.0890	3.3121	0.1710	0.0404	0.8793	-14.3460	0.9031
